# OrbiTox: a visualization platform for NAMs and read-across exploration of multi-domain data

**DOI:** 10.3389/fphar.2025.1710864

**Published:** 2025-12-01

**Authors:** Austin Ross, Vijay Gombar, Alexander Sedykh, Adrian J. Green, Alexandre Borrel, Brian Kidd, Jason Phillips, Mihir Shah, Dhiral Phadke, Deepak Mav, Michelle Balik-Meisner, Brian Howard, Ruchir Shah, Nicole C Kleinstreuer, Warren Michael Casey

**Affiliations:** 1 Sciome LLC, Research Triangle Park, NC, United States; 2 Division of Translational Toxicology at National Institutes of Environmental Health Sciences, Research Triangle Park, NC, United States

**Keywords:** web application, read, across, new approach methodologies, non animal methods, quantitative structure–activity relationship, computational toxicology

## Abstract

The development of new approach methodologies (NAMs) for next-generation risk assessment (NGRA) requires the integration of diverse data streams. A comprehensive understanding of a chemical’s potential hazard involves combining multiple mechanistic data, usually from *in vitro* and *in silico* studies, to build a coherent weight-of-evidence case. Currently, the lack of tools to effectively aggregate and navigate disparate datasets makes regulatory evaluation a challenging process. OrbiTox addresses this need by consolidating millions of data points from multiple domains, i.e., chemical properties, genes, pathways, and bioactivities, into an intuitive and interactive 3D visualization platform. To support comprehensive chemical assessments, OrbiTox incorporates hundreds of quantitative structure–activity relationship (QSAR) models for robust gap-filling of key endpoints. It also facilitates read-across by enabling the retrieval of data-rich chemical analogs with similar structures and metabolic profiles. By unifying experimental data and predictive models within a user-friendly interface, OrbiTox facilitates data-driven chemical safety assessments.

## 1 Introduction

The field of toxicology is undergoing a paradigm shift, moving from traditional animal testing toward the adoption of new approach methodologies (NAMs) (National Toxicology Program, 2018). Modern toxicological studies and next-generation risk assessment (NGRA) now rely on integrating extensive datasets from non-animal testing, such as *in vitro* assays, organs-on-chip, or transcriptomics datasets, to elucidate complex mechanistic pathways in approaches such as integrated approaches to testing and assessment (IATA) and read-across (Barrero-Canosa et al., 2025; Caloni et al., 2022; Roe et al., 2025).

Valuable data for these assessments are housed in disparate repositories such as PubChem (Kim et al., 2016), DrugBank (Wishart et al., 2018), ChEMBL (Gaulton et al., 2017), and GenBank (Benson et al., 2013), but this information is often siloed, stored in heterogeneous formats, and of variable quality. While existing platforms, such as the EPA CompTox Chemicals Dashboard (Williams et al., 2017), provide access to substantial information, they can be overwhelming and do not fully support the comprehensive exploration or integration of all necessary multi-source data. Consequently, a significant gap remains for a unified platform capable of integrating these multi-domain datasets.

We fill this gap by building a translational discovery platform, OrbiTox, with the ability to house and interactively visualize large amounts of multi-domain data and to extract novel knowledge from the connections across these diverse data types. Furthermore, OrbiTox is enriched with predictive models to fill data gaps for untested chemicals.

Access to multi-domain data, predictive models, and cheminformatics methods in one software tool can make it easier to answer questions such as: what are the property profiles of chemicals similar to a specific structure of interest? What are the closest analogs of the chemical of interest, and what is their predicted and experimental toxicity profile? Which chemicals are likely to be active or inactive on a given target of interest for a particular toxicity?

## 2 Methods

### 2.1 Data content

Data in OrbiTox have been retrieved from publicly available resources and carefully cleaned and curated while retaining information on the sources. These are structured into interconnected, concentric ‘orbits’ designed for navigation. From the exterior toward the center of the navigation view, each orbit’s description is listed below.

The *chemistry orbit* houses ∼900,000 chemical substances and their names, SMILES, various identifiers with source links, macro classes, chemical size parameters, etc. A variety of cleaned and harmonized experimental toxicity data from different public sources are associated with a large number of these compounds, e.g., bacterial mutagenicity with and without S9 in OECD-recommended strains (∼6,000 compounds with ∼44,000 measurements), rodent and human carcinogenicity (∼1,300 compounds with ∼1,800 labels), oral, inhalation, and dermal acute toxicity (∼8,000 compounds with ∼16,000 values), ocular irritation (∼400 compounds with ∼700 labels), skin sensitization (∼1,000 compounds with ∼1,800 values), and outcome in Tox21 and ToxCast assays (∼9,000 compounds with over 500,000 readouts). These substances are further categorized to make filtering and focusing on a desired set of chemicals easier, such as Tox21 chemicals (∼9,000), drugs (∼4,500), purine bases (∼7,000), steroids (∼6,500), and PFAS compounds (∼11,000).

The *gene orbit* contains gene names, synonyms, and chromosomal locations from the NCBI for ∼41,000 human genes. These genes have ∼1.7M connections, including linkages to chemicals (e.g., *in vitro* target assays) and other gene targets (e.g., protein–protein interactions).

The *pathway orbit* contains approximately ∼2,000 annotated pathways with the names of their member genes. There are ∼44,000 connections between the pathway and gene orbits.

The *organism orbit* is populated with various *in vivo* toxicity studies representing ∼170 test organisms (or systems), including their genus, family, and life span. There are ∼80,000 connections to the chemical orbit.

### 2.2 Data organization and connections across orbits

Objects in each orbit are clustered based on their within-orbit similarity. Chemicals are clustered in the chemistry orbit by computing their pairwise Jaccard similarity using *Saagar* fingerprints (Sedykh et al., 2021). The gene orbit contains genes clustered based on their gene co-expression from gene-level raw read counts for 441,356 human transcriptomic samples meeting a pre-defined criterion from the ARCHS4 portal (Lachmann et al., 2018). Gene co-expression was quantified based on the pairwise Pearson correlation coefficient (PCC) among the normalized raw read counts. Pathways are collections of annotated pathways taken mainly from KEGG, BIOCARTA, and REACTOME databases (Broad Institute, 2025) and are clustered based on the similarity of gene membership within each pathway. Organisms are organized in the organism orbit based on inter-organism distances defined by the phylogeny tree. The order of the orbits is loosely based on the complexity of their objects, thus placing more complex objects (organisms) closer to the center and chemical structures in the outermost orbit.

Connections between orbits are visualized based on defined criteria, such as experimental data from assays or established biological relationships. For instance, a chemical is linked to the ‘gene’ orbit if it exhibits activity against that gene target. A gene is connected to the ‘pathway’ orbit if it is a known member of that pathway. Finally, an organism is linked to a chemical when corresponding bioactivity data are available (such as toxic effects or phenotype change).

### 2.3 QSAR models

To enrich OrbiTox, the current version offers over 150 robust and cross-validated QSAR models. These models serve as *in silico* NAMs to extend existing experimental data connections with computational predictions. These include models for Tox21 assays (at 100 μM and 10 μM concentration thresholds), bacterial mutagenicity models for predicting outcome in the Ames test conducted using five OECD-recommended strains both in the presence (+S9) and absence (-S9) of metabolic medium, models based on the ToxCast data to predict failure modes of cardiotoxicity, models based on the carcinogenicity data available from the NTP technical reports and other regulatory agencies, and a rat oral TD_50_ model for nitrosamines. Along with predictions from these QSAR models, predictions of metabolites of a compound are also made using SyGMA (Ridder and Wagner, 2008). The training sets have been thoroughly prepared and harmonized to concisely define every modeled endpoint to comply with the OECD “Principle 1” for acceptance of the QSAR model for regulatory applications (OECD, 2014). Models were built following the best practices in the field (Fourches et al., 2010). *Saagar* molecular descriptors were chosen over ToxPrint and Mordred based on their performance in a benchmarking exercise on classification QSAR modeling (Supplementary Figure S1).

## 3 Application description

**FIGURE 1:**
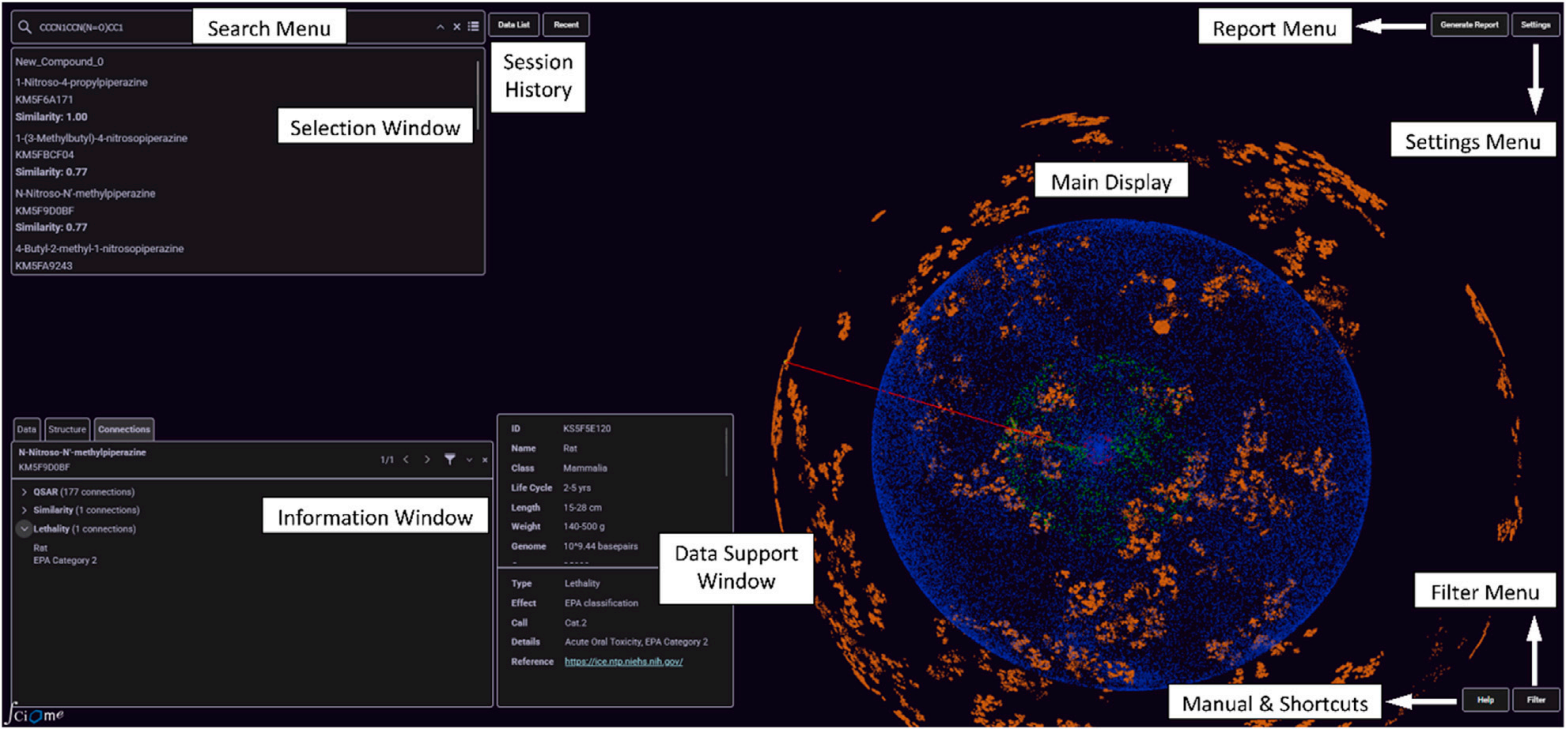
Schematic of the OrbiTox interface.

**FIGURE 2:**
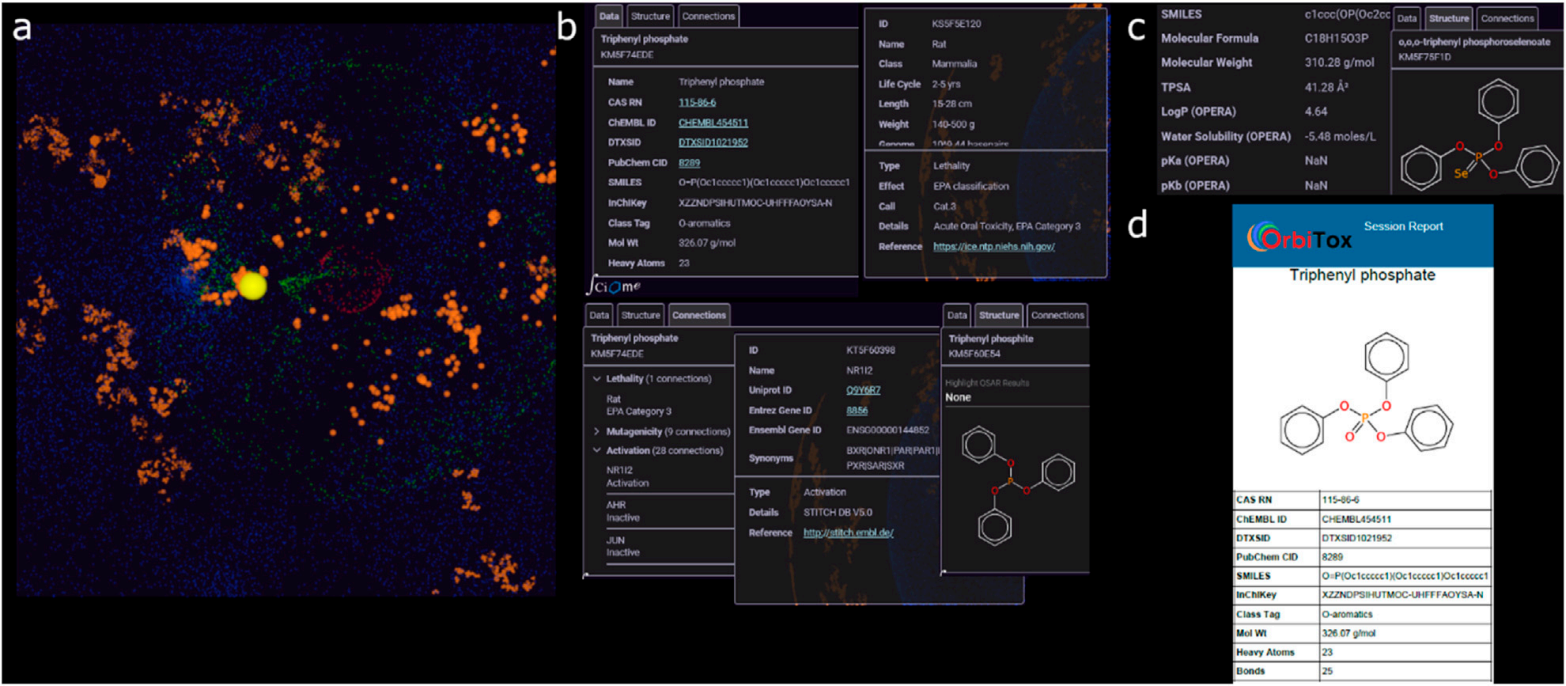
**(a)** Organization of multi-domain data in OrbiTox clustered in concentric orbits: chemicals (orange), gene targets (blue), biological pathways (green), and test organisms (red); **(b)** source data, structural visualization, and experimental results of a compound available in OrbiTox; **(c)** for a new query compound, its computed physicochemical properties and most similar structure in OrbiTox; **(d)** generated report including chemical information, structural analogs, predicted toxicities, and read-across results.

*OrbiTox interface*: The user interacts with OrbiTox through a simple 3D interactive interface of menus and windows (Figure 1), which is designed for high-performance searching, filtering, and exploration of millions of multi-domain data points, enabling real-time visualization with instantaneous updates. Main display (Figure 2a) consists of interconnected chemistry (orange), gene (blue), pathways (green), and organism (red) ‘orbits.’ The search menu accepts user queries for efficient processing and provides robust features such as fuzzy matching and autocompletion. The filter menu controls objects in the main display that meet criteria defined by a user-selected combination of textual and numerical constraints. The settings menu allows customization of node attributes, including size, shape, and color.

*OrbiTox Operation*: After invoking OrbiTox (www.orbitox.org), the user enters the name/ID/SMILES of a compound, a gene, a pathway, or an organism in the search window, depending on the use case of interest. OrbiTox identifies the selected object in the appropriate orbit and moves focus to the chosen object. On selecting an object, the information window with three tabs (structure, data, and connections) is populated with relevant information. For an acceptable 1D of a chemical as a query, for example, OrbiTox allows a user to interactively visualize it (red dot, Figure 1) in the vicinity of chemicals with similar structures. The content in the connection tab provides information on its experimental data (toxicity, ADME (absorption, distribution, metabolism, excretion), and pharmacology) and biological targets and organisms it interacts with (Figure 2b). If the query is a SMILES string of a chemical, OrbiTox recognizes it as a new chemical and makes predictions of outcomes in hundreds of bioactivity assays with QSAR models that provide chemistry-backed reasoning for each prediction (Figure 2c). A customized, printable, or machine-readable report (Figure 2d) can be generated from the Report Menu.

The user can easily navigate through various menus and options of the user-friendly interface of OrbiTox to conduct desired investigations for objects from any of the four connected domains. Several common applications of OrbiTox, useful in an NGRA process, are listed below.

1. Extract all experimental data available for a query chemical (query entered as the chemical’s name or acceptable 1D).
2. Generate a property profile of a chemical based on over a hundred validated QSAR models (query entered as a SMILES string).
3. Visualize data-rich compounds with structures similar to that of the query (the number and similarity distance of similar structures are customizable).
4. Visualize structural features that are responsible for differences in properties of compounds despite being similar in structure.
5. Analyze why, despite similarity in structures, two chemicals have different property profiles.
6. Find correlation between data for a set of compounds tested in two different biological assays for efficient screening, e.g., selecting an *in vitro* assay over an animal assay.
7. Collect data for modeling with compounds that interact with a gene target, such as PPARD, or that have been tested in a particular organism, e.g., *Salmonella Typhimurium* strain TA100.
8. Identify member genes of a pathway to find potential new therapeutic targets, such as genes BECN1 and ATG5 of the autophagy pathway as targets of breast cancer treatment.
9. Conduct a read-across assessment by identifying an analog with similarity in structure and similar physicochemical, biochemical, and metabolic profiles (Figure 1), e.g., N-nitroso-N′-methylpiperazine as a source analog for 1-nitroso-4-propylpiperazine (SMILES, CCCN1CCN(N=O)CC1).
10. Assess potential liabilities before advancing a nanomolar pharmacologically active hit to plan testing strategies, e.g., test Ranirestat (inhibitor of AKR1B1 with IC_50_ = 15 nM) in a mitochondrial pathway activation assay (predicted activator with probability 0.832).

## 4 Conclusion

OrbiTox is a web-based translational discovery platform (https://orbitox.org) that enables multi-domain data exploration within an interactive 3D environment. By facilitating connections between data from different domains, the platform opens novel avenues for NGRAs. In addition, OrbiTox uniquely integrates diverse bioactivity data from both *in vivo* and *in vitro* sources. Direct comparison of animal and non-animal data is important for building confidence in and establishing the relevance of NAMs, ultimately supporting the transition to animal-free safety testing. Finally, the OrbiTox architecture may be expanded to incorporate more domains, data, and QSAR models, including proprietary data and models.

## Data Availability

Publicly available datasets were analyzed in this study. This data can be found here: https://orbitox.org.

## Supplementary material

SUPPLEMENTARY FIGURE S1 Average AUROC for the predictions of left-out compounds in triplicate 5-fold cross-validation by different modeling methods using Saagar, ToxPrint, and Mordred descriptors for the Ames bacterial mutagenicity QSAR models. Saagar performs better or as good as ToxPrint and Mordred in different modeling methods.
