# Supplementary figures and images for: OrbiTox: a visualization platform for NAMs and read-across exploration of multi-domain data
Source: Front Pharmacol. 2025 Dec 1;16:1710864. doi: 10.3389/fphar.2025.1710864 (PMC12702942; doi:10.3389/fphar.2025.1710864)

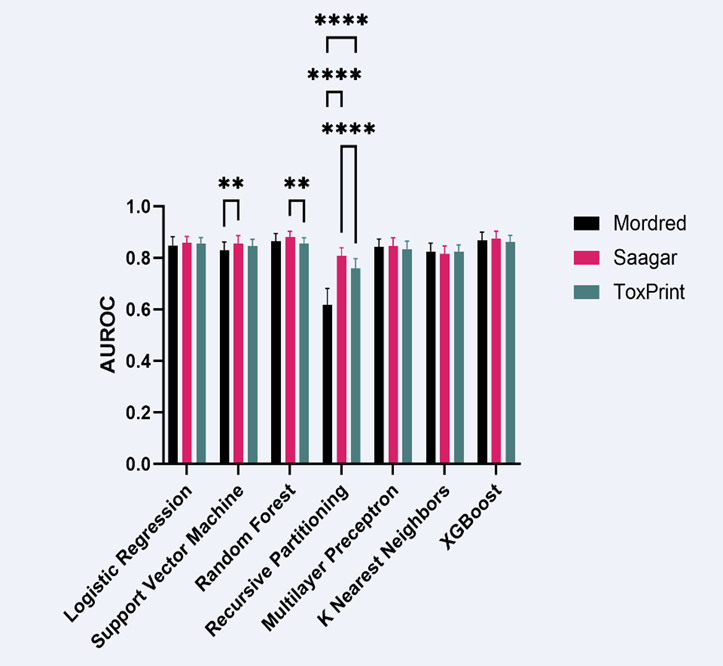

Supplement: Supplementary file 1 [file Image1.tiff]
